# Innovative feedstocks for optimal mass production of the edible long-horned grasshopper, *Ruspolia differens*

**DOI:** 10.3389/fphys.2022.1015636

**Published:** 2022-11-09

**Authors:** Kababu Margaret, Mweresa K. Collins, Sevgan Subramanian, James P. Egonyu, Dorothy Nakimbugwe, Geoffrey Ssepuuya, Nyamu Faith, Sunday Ekesi, Chrysantus M. Tanga

**Affiliations:** ^1^ International Centre of Insect Physiology and Ecology (icipe), Nairobi, Kenya; ^2^ School of Agricultural and Food Sciences, Jaramogi Oginga Odinga University of Science and Technology (JOOUST), Bondo, Kenya; ^3^ Department of Food Technology and Nutrition, School of Food Technology, Nutrition and Bioengineering, Makerere University, Kampala, Uganda

**Keywords:** edible grasshoppers, *Ruspolia differens*, low-cost artificial diets, growth performance, fecundity, food security

## Abstract

The edible long-horned grasshopper *Ruspolia differens* Serville (Orthoptera:Tettigoniidae) is a highly nutritious food source consumed in over 20 African countries. Its occurrence is highly seasonal, and wild harvesting is carried out using locally designed and inefficient light traps, thus limiting sustainable utilization as an important food source. To ensure year-round production and availability of *R. differens*, we evaluated the effects of low-cost and affordable diets based on agricultural by-products on their growth performance, survival, fecundity, and longevity. A total of four diets with varying ratios of agricultural by-products were evaluated: Diet 1 [33.3% maize bran (MB) + 33.3% wheat bran (WB) + 33.3% *Moringa oleifera* leaf powder (MOLP)], Diet 2 [25% MB + 25% WB + 25% MOLP + 25% shrimp powder (SP)], Diet 3 [20% MB + 20% WB + 20% MOLP + 20% SP + 20% soya bean meal], and Diet 4 (“control”—routinely used diet). The grasshoppers were subjected to the diets from the 1st nymphal instar (24-h-old stages) through adult stages until death. Diet 3 had the highest crude protein content (28%) and digestibility (74.7%). *R. differens* fed Diet 3 had the shortest development time (57 days) [*p* < 0.001], highest survival (87%) [*p* < 0.001], and maximum longevity (89 days) [*p* = 0.015] and fecundity (247 eggs/female) [*p* = 0.549] across the various diets. Female survival rate (59%) on Diet 3 was significantly higher compared to the males (41%). The adult female weight gain was significantly higher compared to males fed on different diets. Percentage hatchability of eggs was not significantly different when females were fed Diet 3 and Diet 2. There was a significantly positive correlation between longevity and fecundity of *R. differens* reared on Diet 2 and 3. These diets could be further optimized and fine-tuned for improved cost-effective mass production of *R. differens* continent-wide to reduce dependence on erratic and poor seasonal harvest during swarms.

## 1 Introduction

*Ruspolia differens* Serville (Orthoptera: Tettigoniidae) is one of the popularly consumed insects in Eastern and Southern Africa (Kinyuru et al., 2010; van Huis et al., 2013). The grasshopper contains high levels of proteins, fats, vitamins, and minerals that are essential for human nutrition (Kinyuru et al., 2010; Mmari et al., 2017). It can serve as a suitable alternative for the rising demand for animal proteins and increasing cases of malnutrition (van Huis, 2013; Paul et al., 2016). It has a high market value, with prices comparable to beef and chicken, and serves as a source of livelihood for many households (Mmari et al., 2017; Okia et al., 2017). Despite this, the consumption and utilization of *R. differens* as a market commodity is limited by seasonality because it is only harvested from the wild during the long (April–May) and short (November–December) rainy seasons when they swarm (Agea et al., 2008; Okia et al., 2017).

The need for domestication of *R. differens* to enhance sustainable production and utilization has been expressed (Valtonen et al., 2018). However, successful domestication and production of such insects require optimization of appropriate conditions including quantity and quality of feed (van Huis, 2013; Dobermann et al., 2017). In the laboratory, *R. differens* is known to feed on fresh grasses and sedges, which are locally threatened by a lack of sustainable conservation practices (Okia et al., 2017; Ssepuuya et al., 2018; Valtonen et al., 2018; Malinga et al., 2020). Some diets including food crops such as rice, finger millet, and sorghum seed heads have been evaluated for rearing the insect, but they are also consumed by humans and their use as feed for the grasshoppers may be a threat to the rising food insecurity (Malinga et al., 2018; Sorjonen et al., 2020). Other diets are based on processed animal feed such as dog biscuit pellet, chicken super feed egg booster, and starter chicken feed, which are expensive and inaccessible (Malinga et al., 2018; Ssepuuya et al., 2018). Thus, agricultural and food industry by-products present a potentially cheap and sustainable feed source for the production of edible insects and have been used to feed crickets and grasshoppers (Miech et al., 2016; Sorjonen et al., 2020). Such by-product feed for insects should meet the nutritional requirements, allowing for maximal weight gain, high feed efficiency, faster development, and high survival of target insects (Sorjonen et al., 2020). Meanwhile, diets comprising purely agricultural by-products contribute a crude protein level of 24% and an average survival of 34% in crickets, and supplementation of agricultural by-products with conventional feed resulted in crude protein content of 23% and an average survival of 60% (Kuo and Fisher, 2022). However, there is limited data on the use of agricultural by-products as a feed source for *R. differens*. Sorjonen et al. (2020) demonstrated increased growth, survival, and development rate when *R. differens* were reared on agricultural by-products. However, the diets were based on agricultural by-products that are not readily available in the Eastern Africa region where the grasshopper is predominant.

Limited data exist on the use of *Caridina nilotica* P. Roux (Decapoda: Atyidae) and *Moringa oleifera* Lam (Brassicales: Moringaceae) in formulation on *R. differens* feed. *Caridina nilotica* is a bycatch of the silver cyprinid fishery in Lake Victoria. It is underutilized in Kenya where it is mainly incorporated in animal feed due to its high protein content (Mugo-Bundi et al., 2015; Kubiriza et al., 2018). *Moringa oleifera* is highly nutritious and rich in vitamins, minerals, and essential amino acids; it has higher digestibility and improves feed efficiency in animals (Moyo et al., 2016; Abbas et al., 2018; El-hack et al., 2018). Maize bran (*Zea mays* Linnaeus) (Poales: Poaceae), wheat bran (*Triticum aestivum* Linnaeus) (Poales: Poaceae), and soybean meal [*Glycine* max (L.) Merr] (Fabales: Fabaceae) have previously been used in production of *R. differens* (Malinga et al., 2018b, Malinga et al., 2020). Maize and wheat bran are agro-industrial by-products of maize and wheat processing, respectively, with high crude protein content and are used for formulation of feed (de Groote et al., 2010; Mutayoba et al., 2011; Mwesigwa et al., 2012). Soybean, on the other hand, is produced for human and animal consumption and is increasingly used as a protein source for animal feed globally due to its high protein content (Dei, 2011; Mukherjee et al., 2016).

The rearing performance of *R. differens* on different artificial diets remains sub-optimal with long nymphal development time, high mortality, and low fecundity (Malinga et al., 2018; Leonard et al., 2022). Therefore, there is a need to formulate a nutrient-dense compound feed from readily available diets with a long shelf life for rearing of *R. differens*. The effects of the formulated diets on growth, development, and reproductive performance of *R. differens* should be determined to inform domestication and mass production (Ssepuuya et al., 2018). This study was aimed at formulating low-cost diets derived from agricultural by-products and assessing their effects on the growth, survivorship, longevity, and reproductive performance of *R. differens.* In addition to high protein content, the diets were selected because they are cheap, locally available, and accessible in the study region. Acceptance and preference of different diets by *R. differens* were assessed prior to formulation of diets. The chemical and nutritional compositions of the diets were also determined, and their unit cost was estimated.

## 2 Materials and methods

### 2.1 Rearing of *Ruspolia differens* colony for experiments

The *R. differens* used in this study were obtained from the 8th generation of the existing colony at the International Centre for Insect Physiology and Ecology (*icipe*) Duduville Campus in Nairobi, Kenya. The colony was established in November 2017 using grasshoppers collected from commercial traps in Uganda. The grasshoppers were reared in Perspex cages measuring 50 × 50 × 50 cm placed in a rearing room maintained at 27°C–30°C, 50%–65% RH, and a photoperiod of 12:12 h of day:night. The *R. differens* were fed mainly on a powdered artificial diet formulated by Treasure Feeds Limited (Thika, Kenya) (Egonyu et al., 2021). The diet was supplemented with fresh shoots of *Panicum maximum* Jacq (Poales: Poaceae) and *Brachiaria ruziziensis* Germ & C.M Evrard (Poales: Poaceae). The artificial diet was presented in Petri dishes, whereas fresh shoots from both plant materials were presented as bouquets in clean plastic containers (height 11 cm and diameter 5 cm) half filled with water and secured at the surface with cotton wool, respectively. This prevented the plants from withering and the insects from drowning in water while feeding (Egonyu et al., 2021). In total, three Petri dishes containing the specific diet and three bottles of plant materials were placed in each cage. The plant materials were replenished after every 3 days. Water was provided through soaked cotton balls, which were also used as oviposition substrates. The eggs were collected by opening the cotton balls and unsheathing the plant materials that contained eggs. The collected eggs were placed on moist cotton wool in 2-L rectangular plastic containers measuring 220 × 156 × 82 mm (Rectangle Food Mate No. 2, Kenpoly Manufacturers Limited, Nairobi, Kenya) with ventilation at the top. Water was sprinkled on the eggs daily until hatching to prevent desiccation. The containers were placed on a table within the rearing room and monitored regularly for nymphal hatching.

### 2.2 Experimental diets

#### 2.2.1 Source of diet substrates and processing of diets

A total of five diet substrates, namely, maize bran, wheat bran, lake shrimps (*C. nilotica*; local name *ochong’a*), *M. oleifera* leaves, and soybean meal were selected for formulation of experimental diets. Maize bran, wheat bran, soybean meal (powdered), and *ochong’a* were purchased from a vendor at a local market (Gikomba market, Nairobi) and ground into fine powder (0.01–0.02 mm) using an electronic grinder (Preethi TRIO, 500w, MG182/00, Preethi Kitchen Appliances Pvt, Ltd. Chennai, India). *Moringa oleifera* leaves were acquired in a dried and powdered form from a farmer from Bondo County.

#### 2.2.3 Assessment of the acceptance and preference of the diet components

Acceptability and preference of five diet substrates by *R. differens* were tested using no-choice and choice bioassays, respectively. In the no-choice test, a total of 10 adult grasshoppers were collected from the wild and presented with 2 g of each of the feed substrate for a period of 48 h. Each grasshopper was held in a ventilated 4-L transparent plastic container where they had *ad libitum* access to feed and water. The diets were presented on a piece of aluminum foil, and water was provided in a soaked cotton ball. The feed residue was weighed after 48 h. The quantity of feed substrate consumed was determined by subtracting the amount of feed residue from the initial feed provided (Orinda et al., 2017).

In the choice experiment, each of the 10 grasshoppers used in the no-choice test were presented with 2 g of the five diets placed side by side for a period of 48 h. Each grasshopper was placed in a ventilated 4-L transparent plastic container. The diets were presented on a piece of aluminum foil, and water provided in a soaked cotton ball. The quantity of each of the diets left was weighed after 48 h. The quantity of feed substrate consumed was determined by subtracting the amount of feed residue from the initial feed provided (Orinda et al., 2017).

#### 2.2.4 Formulation of diets

A total of three diet mixtures were constituted using an equal proportion (100 g) of the five ingredients in an increasing gradient of three to five based on their preferential selection by *R. differens* (Table 1). The diet mixtures were stored in clean transparent plastic containers and placed in a cupboard. The artificial diet mixture used in the rearing of *R. differens* at the International Centre for Insect Physiology and Ecology (icipe) was used as control (Egonyu et al., 2021). The control diet comprised bergafat, maize flour, lysine amino acid, vitamin mineral premix, dicalcium phosphate (DCP), lime, silver cyprinid (local name: *omena*), salt, wheat pollard, rice polish, soya meal, and methionine (Treasure Feeds Ltd., Thika, Kenya).

**TABLE 1:** Composition of the diets used to assess development, survivorship, longevity, and reproductive performance of *Ruspolia differens*.

Diet	Composition in percentages				
	Maize bran	Wheat bran	MOLP	*Ochong’a*	Soybean
Diet 1	33.3	33.3	33.3	0.0	0.0
Diet 2	25.0	25.0	25.0	25.0	0.0
Diet 3	20.0	20.0	20.0	20.0	20.0
Diet 4 (Control)	Bergafat, maize flour, lysine amino acid, vitamin and mineral premix, dicalcium phosphate (DCP), lime, silver cyprinid (local name: *omena*), salt, wheat pollard, rice polish, soybean meal, and methionine				

#### 2.2.5 Nutritional analysis of the experimental diets

A nutritional analysis of the diets was conducted at ILRI laboratory. Dry matter, crude fat, crude protein, crude fiber, ash, ADF (acid detergent fiber), and NDF (neutral detergent fiber) contents of the diets were determined using the official methods of Association of Official Analytic Chemists (AOAC, 2012). Sugar and starch content of the diets were computed using NIR technology (Lebot et al., 2009). Protein digestibility of the diets was determined using a modified method described by Mertz et al. (1984) using pepsin enzyme. An inductively coupled plasma–optical emission spectrometer (ICP-OES) was used to determine the mineral composition of study diets (Campbell and Plank, 1991; Horwitz, 2000).

#### 2.2.6 Cost of the experimental diets

The unit cost of each of the experimental diets was computed in MS Excel. The cost was generated by calculating the quantity of individual ingredients, which constated a kilogram of each of the experimental diets. This was then multiplied by the market price for a kilogram of respective ingredients, which were summed up to provide the cost of a kilogram of each of the experimental diets.

### 2.3 Experimental method

#### 2.3.1 Experimental design

A completely randomized design was used with four diet treatments, namely, Diet 1, Diet 2, Diet 3, and Diet 4 (Table 1), by three replicates. The experiment was set up in a laboratory maintained at 27°C–29°C, 50%–60% RH, and a photoperiod of 12:12 h day:night. In total, 30 newly emerged nymphs of *R. differens* were reared in ventilated Perspex cages measuring 40 × 40 × 40 cm. Each treatment was assigned three cages (three replicates). The cages were placed on shelves elevated at 1 m above the ground. All the cages were placed 15 cm away from the wall and 10 cm away from each other.

#### 2.3.2 Effect of diets on wet weight, development, and survivorship of *R. differens*

A total of 30 newly hatched (1–2 days) nymphs were released into each of the experimental cages to assess the effects of experimental diets on development and survivorship of *R. differens*. The nymphs were released by placing uncovered incubation containers into a clean cage at the onset of egg hatching. The process was repeated until the desired number of nymphs was attained for each of the experimental cages. The nymphs had *ad libitum* access to food and water. In total, 5 grams of each of the diets were presented in a Petri dish and replaced every week. The quantity of diet was deemed adequate to feed the nymphs with minimal wastage, since the leftover diet was replaced on a weekly basis. Water was provided through soaked cotton balls, replaced after every 3 days. The cages were cleaned at 3-day intervals. The cages were monitored for mortality on daily basis. A total of 10 randomly selected nymphs were weighed weekly, while adults were weighed within 24 h of emergence using an electronic weighing scale (Model kern PCB 350-3) and their sex was determined. The sexes were differentiated by the presence of the ovipositor in females (Matojo and Yarro, 2013) The nymphs were placed in a transparent plastic container (12 × 6 cm). and weight measurement readings on the scale were taken once there was no movement of the grasshoppers. The weight of the grasshoppers was logged as the total weight minus the container weight (Magara et al., 2019). Development time was determined as the days between hatching and adult molt (Malinga et al., 2020). Survival was estimated by calculating the number of individuals alive at the end of the experiment (Sorjonen et al., 2019).

#### 2.3.3 Reproductive performance and adult longevity of *R. differens*

**FIGURE 1:**
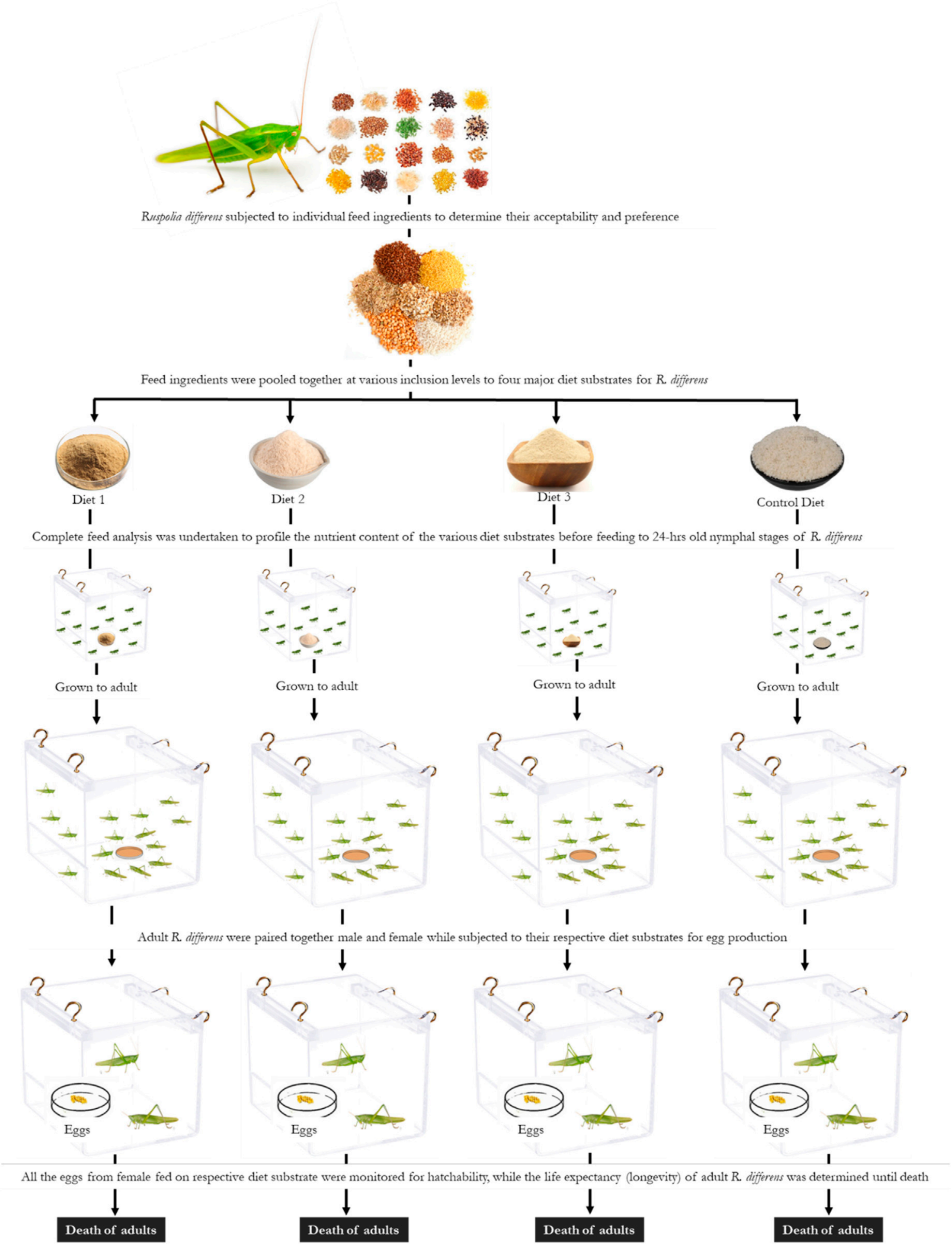
Schematic representation of the experimental set up of *Ruspolia differens* fed on four different diet substrates.

In order to determine the effects of diets on reproductive performance and longevity, 10 pairs (male and female) of newly molted adult *R. differens* were used for each of the diets and monitored until death. The paired insects were placed in transparent 4-L plastic containers measuring 220 × 156 × 160 mm (Rectangle Food Mate No. 3, Kenpoly Manufacturers Limited), placed randomly on shelves in the laboratory. The insects were maintained under similar conditions and feeding regimes described previously. When cleaning and changing feed, the position of containers were randomized to avoid potential shelf effects (Lehtovaara et al., 2018). Eggs collected from each female were incubated in 1-L cylindrical plastic containers (Thermopak TPL 2033; Thermopak Limited, Nairobi, Kenya), which were ventilated with a plastic mesh at the top. The eggs were spread on wet cotton wool and sprinkled with water every morning to moisten the cotton wool and prevent desiccation. The containers were monitored daily for freshly hatched nymphs, and the numbers were recorded until no more hatching occurred. Pre-oviposition time was calculated from the day of emergence of females to the first day of oviposition (Magara et al., 2019). Fecundity was determined as the total number of eggs laid per female (Malinga et al., 2018b). Egg incubation was calculated from the day of egg oviposition to hatching. Hatchability was calculated as a percentage of eggs hatched/total eggs laid per female (Roy and Barik, 2012b). Duration of egg eclosion was determined from the onset of hatching to the last day when hatching occurred. Post-oviposition time was calculated as the time between the oviposition of last egg and the death of female adult. Adult longevity was calculated from the day of adult emergence and its death. A schematic flow of the experimental set up is provided in Figure 1.

### 2.4 Statistical analysis

All data were subjected to Shapiro–Wilk and Bartlett tests prior to analysis. A non-parametric Kruskal–Wallis one-way analysis of variance (ANOVA) was used to test whether food acceptance differed amongst the diets. Differences in means were separated using a pairwise Wilcoxon test with BH correction. Differences in ranks in food preference were determined using Friedman’s ANOVA, which is a non-parametric alternative to unreplicated block design ANOVA. A pairwise Wilcoxon test with Bonferroni correction was used to separate the means where the differences were significant. The differences in the survival rate of *R. differens* nymphs to adult molt and percentage hatchability of eggs between diets were analyzed using a logit-linked binomial generalized linear model (GLM). Differences in pre-oviposition time, oviposition duration, post-oviposition time, fecundity, duration of egg incubation, and egg eclosion time among diets were determined using a negative binomial GLM, which was used to correct over dispersion that occurred when the data were subjected to a log-linked Poisson distribution. One-way analysis of variance (ANOVA) was used to analyze data on weight, development time, and male adult longevity. Tukey’s honest significant difference (HSD), a *post hoc* test, was used as a multiple-comparison procedure to test where categories differed significantly. Data on female longevity were analyzed using Welch ANOVA. A pairwise Wilcoxon test was used to separate the differences in means where they differed significantly. A correlation test was used to determine the association between weight and fecundity, fecundity and longevity, and oviposition duration and longevity. All effects were considered significant at *p* < 0.05. All analyses were performed using R version 4.1.2 (R Core Team, 2020) statistical software.

## 3 Results

### 3.1 Acceptability of diet substrates

**FIGURE 2:**
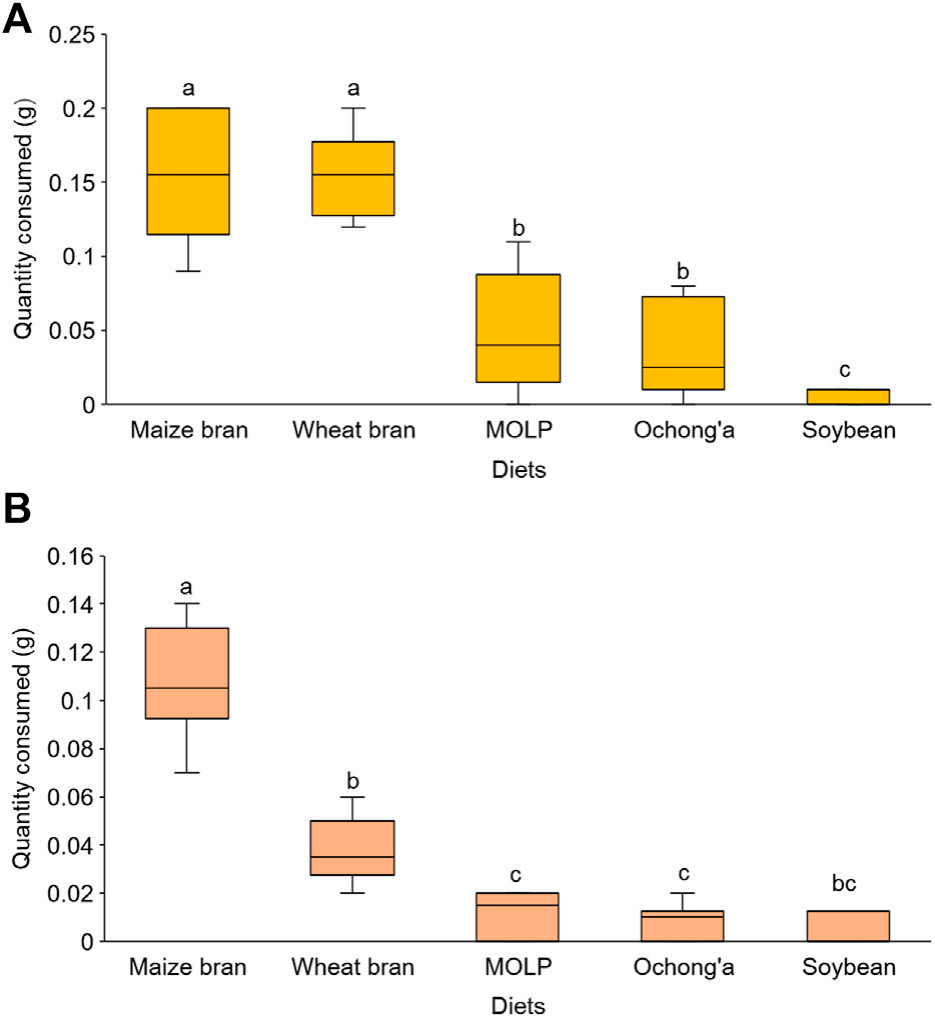
**(A)** Acceptance and **(B)** preference of different diet substrates by *Ruspolia differens* ranging from the most to the least preferred. Box plots capped with different letters differed significantly at *p* < 0.05.

In the no-choice experiment, all the five diet substrates were accepted by *R. differens* in diverse quantities. The acceptance of the diets differed significantly (Kruskal–Wallis chi squared = 34.847, df = 4. *p* < 0.001). Maize and wheat bran were the most accepted, while soybean was the least accepted feed substrate (Figure 2A). There were no differences in the acceptance of maize and wheat bran (*p* = 0.939) and MOLP and *ochong’a* (*p* = 0.781) by *R. differens.*

### 3.2 Preference of diet substrates

In the choice test, feed preference differed significantly among substrates (Friedman’s test *x* ^2^ = 33.29, df = 4, *p* < 0.001). Maize bran was the most preferred followed by wheat bran, Moringa, *ochong’a*, and then soybean (Figure 2B). However, *R. differens*’ preference for MOLP and *ochong’a* (*p* = 1.000), soybean meal and MOLP (*p* = 1.000), soybean meal and *ochong’a* (*p* = 1.000), and soybean and wheat bran (*p* = 0.085) were not significantly different.

### 3.3 Nutritional composition of the diets used for rearing *R. differens*

Diet 1 had the least quantity of micro minerals: iron (184 mg/kg), copper (7.09 mg/kg), zinc (50.3 mg/kg), cobalt (0.1 mg/kg), manganese (64.7 mg/kg), and sodium (51.6 mg/kg). Diet 2 contained the highest quantity of iron (876 mg/kg), copper (21.7 mg/kg), manganese (93.5 mg/kg), and sodium (1,200 mg/kg). Diet 3 had the highest crude protein content (28%), while Diet 4 had the highest carbohydrate (27%) and crude fat (9%) content. A higher digestibility was observed in Diet 3 (74.7%) (Table 2).

**TABLE 2:** Nutritional composition (% DM basis) of diets used for rearing *Ruspolia differens*.

Nutritional composition	Diet			
	Diet 1	Diet 2	Diet 3	Diet 4
Boron (mg/kg)	8.3	7.7	10.9	9.8
Molybdenum (mg/kg)	1.4	1.2	1.5	1.5
Iron (mg/kg)	184.0	876.0	687.0	551.0
Copper (mg/kg)	7.1	21.7	18.8	13.0
Zinc (mg/kg)	50.3	75.9	72.2	95.9
Cobalt (mg/kg)	0.1	0.3	0.4	0.5
Manganese (mg/kg)	64.7	93.5	87.5	82.1
Sodium (mg/kg)	51.6	1,200.0	942.0	776.0
Sulfur (%)	0.4	0.5	0.4	0.3
Magnesium (%)	0.4	0.4	0.3	0.3
Potassium (%)	1.4	1.4	1.4	0.9
Phosphorus (%)	0.7	0.9	0.7	1.0
Calcium (%)	0.5	1.6	1.4	1.0
Carbohydrate (sugar/starch) %	17.0	11.9	11.8	26.9
Crude fat (%)	5.2	4.6	7.1	9.2
Crude protein (%)	16.7	25.3	27.5	21.1
Dry matter (%)	91.8	91.6	91.6	91.4
Ash (%)	6.1	10.0	9.3	8.1
Fiber (%)	11.8	12.3	15.9	3.6
ADF (%)	19.3	16.4	15.9	20.0
NDF (%)	31.6	22.6	21.2	23.8
Digestibility (%)	67.9	72.4	74.7	74.5

ADF, acid detergent fiber; NDF, neutral detergent fiber.

### 3.4 Cost of diets used

Diet 1 was the most expensive (USD 1.06/kg), followed by Diet 3 (USD 0.99/kg), while the control (Diet 4) was the cheapest (USD 0.79/kg) (Table 3).

**TABLE 3:** Cost of diets used for rearing *Ruspolia differens*.

Feed ingredient	Price/kg (KES)	Quantity per kilogram			
		Diet 1	Diet 2	Diet 3	Diet 4
Maize bran	60.00	0.33	0.25	0.20	0.00
Wheat bran	50.00	0.33	0.25	0.20	0.00
*Moringa olifera* leaves powder	250.00	0.33	0.25	0.20	0.00
*Ochong’a*	50.00	0.00	0.25	0.20	0.00
Soya meal	150.00	0.00	0.00	0.20	0.16
Maize	100.00	0.00	0.00	0.00	0.25
Bergafat	250.00	0.00	0.00	0.00	0.05
Lysine amino acid	450.00	0.00	0.00	0.00	0.001
MDCP	600.00	0.00	0.00	0.00	0.01
Methionine	1,250.00	0.00	0.00	0.00	0.01
Vitamin mineral premix	450.00	0.00	0.00	0.00	0.01
Lime	10.00	0.00	0.00	0.00	0.01
*Omena*	175.00	0.00	0.00	0.00	0.15
Salt	35.00	0.00	0.00	0.00	0.01
Wheat pollard	40.00	0.00	0.00	0.00	0.22
Rice polish	30.00	0.00	0.00	0.00	0.15
Cost per kg (KES)		119.88	102.50	112.00	124.64
Cost per kg (USD)		1.06	0.90	0.99	1.10

Note: 1 USD (US dollar) = KES (Kenya shillings) 113.45 during the period of study (2020/2021).

### 3.5 Effect of diets on developmental duration of *R. differens*

The nymphal development period varied significantly among the diets (F _3, 19_ = 9.663, *p* < 0.001) (Table 4). The shortest mean development duration of 57 ± 2.18 days occurred where Diet 3 was used, while the longest occurred for Diet 1 (75 ± 2.06). Male grasshoppers matured faster than females (F _1, 18_ = 9.575, *p* < 0.01). No variations were observed in the development time of male grasshoppers raised on different diets (F _1, 7_ = 3.914, *p* = 0.062); however, there were differences among females (F _1, 8_ = 8.937, *p* = 0.006).

**TABLE 4:** Mean (±SE) development duration of *Ruspolia differens* reared on the different diets.

Diet	Overall developmental period (days)	Developmental period by sex (days)	
		Male	Female
Diet 1	75.4 ± 2.1^a^	70.4 ± 1.9^a^	78.7 ± 0.3^a^
Diet 2	57.2 ± 3.2^b^	54.7 ± 5.0^a^	59.3 ± 3.9^b^
Diet 3	57.2 ± 2.2^b^	52.9 ± 1.0^a^	61.3 ± 2.3^b^
Diet 4	64.5 ± 2.9^ab^	60.8 ± 4.0^a^	68 ± 3.6^ab^
F-value	9.66	3.91	8.94
*Df*	3, 19	3,7	3, 8
*p*-value	<0.00L	0.062	0.006

Note: Mean (±SE) number of days with different superscript letter (s) in the same column are significantly different at *p* < 0.05.

### 3.6 Effects of diets on the nymphal survival rate of *R. differens* to the adult stage

**FIGURE 3:**
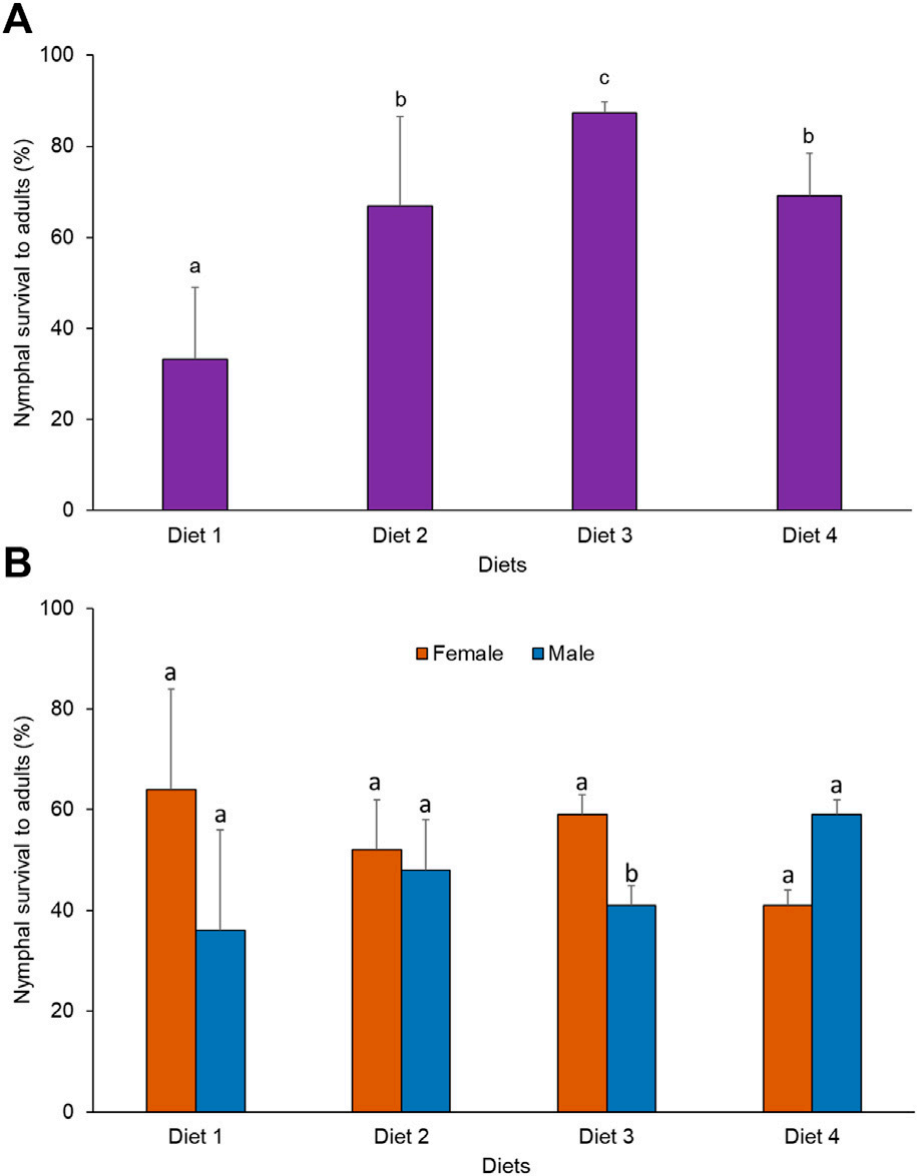
**(A)** Overall survival rate (%) and **(B)** survival rate (%) by sex of *Ruspolia differens* nymphs to adults when reared on different diets. Means capped with different letters differed significantly (Tukey HSD, *p* < 0.05)

A significant difference was observed in the rate of survival of nymphs to adults when *R. differens* was fed on the four diets (*x* ^2^ = 62.184, df = 3, *p* < 0.001). Survival was highest in grasshoppers fed Diet 3 (87%) and lowest for Diet 1 (33%) (Figure 3A). More male *R. differens* survived when fed Diet 4, while higher numbers of females survived when fed Diet 1, 2, and 3 (Figure 3B).

### 3.7 Effect of diet on wet weights of *R. differens*

**FIGURE 4:**
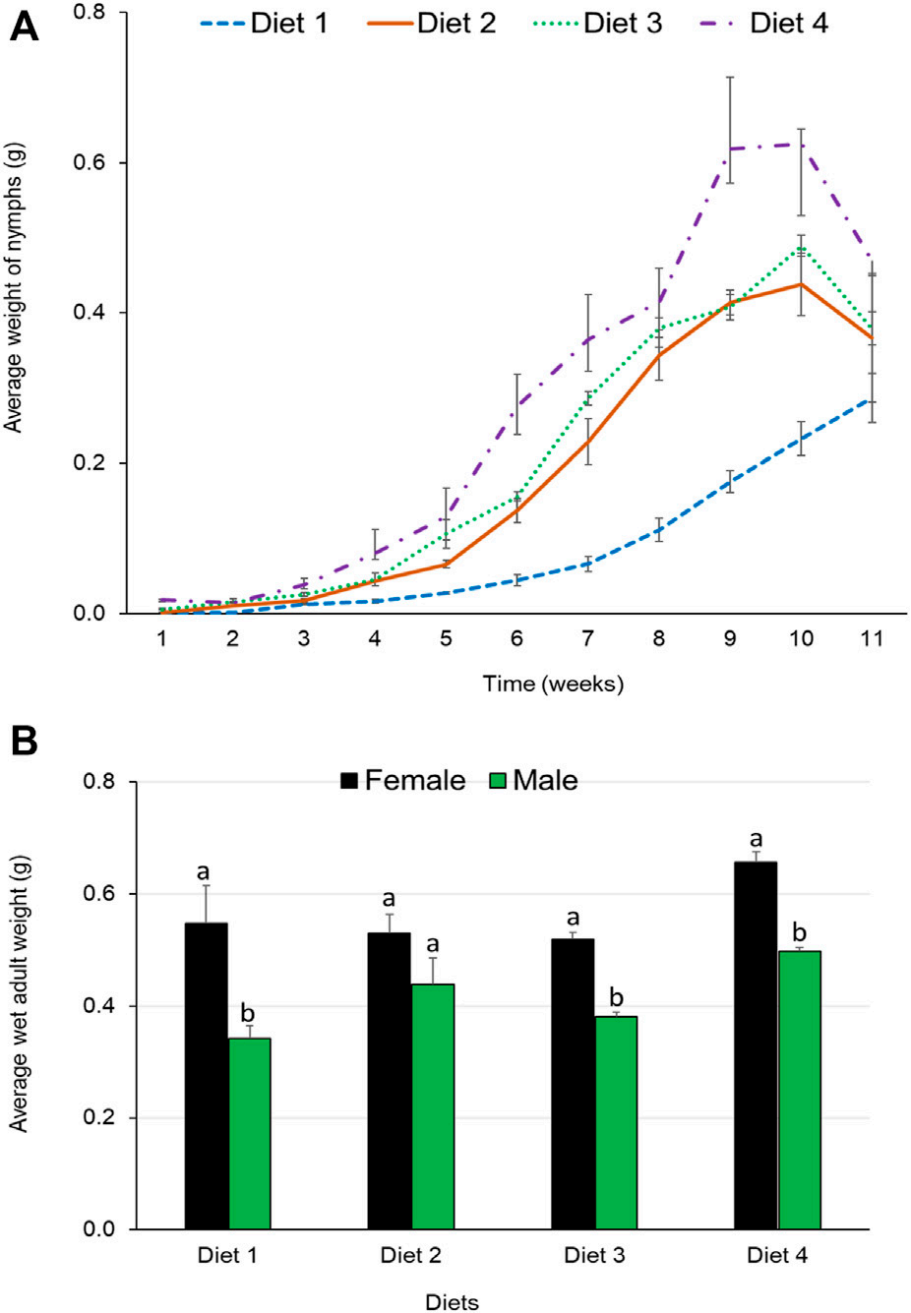
**(A)** Mean weekly wet weights of nymphs and **(B)** mean wet weights by sex of adult *Ruspolia differens* raised on different diets.

*Ruspolia differens* nymphs fed Diet 4 maintained the highest average increase in weight throughout the development period (Figure 4A). However, wet adult weights did not vary significantly among *R. differens* fed on different diets (F _3,19_ = 2.007, *p* = 0.147). Grasshoppers fed Diet 4 had the highest average weight (0.58 g), while those fed Diet 1 had the least weight (0.45 g) (Figure 4B). The wet weight of female grasshoppers was higher than that of males (F _1, 18_ = 34.808, *p* < 0.001). Weight of the male grasshoppers differed among diets (F _3,7_ = 2.007, *p* = 0.016), while there was no difference among the female (F _3,8_ = 2.563, *p* = 0.128). The highest male (0.5 g) and female (0.66 g) weights were recorded in *R. differens* fed Diet 4.

### 3.8 Effect of diet on reproductive performance of *R. differens*

#### 3.8.1 Effect of diet on the pre-oviposition period, duration of oviposition, post-oviposition duration, incubation time, and duration of egg eclosion

Pre-oviposition time differed among *R. differens* raised on different diets (χ^2^ = 11.11, df = 3, *p* = 0.011). The shortest pre-oviposition time occurred in *R. differens* fed Diet 4 (13 days), while longest time was observed in Diet 1 (28 days).

Duration of oviposition (χ2 = 36.59, df = 3, *p* < 0.001) differed significantly among *R. differens* fed the different diets. The longest oviposition duration occurred in *R. differens* fed Diet 3 (54 days), while those fed Diet 2 oviposited over the shortest period (36 days) (Table 5). A positive significant correlation was observed between oviposition duration and longevity in Diet 2 (Spearman’s rho = 0.89, *p* < 0.001) and Diet 3 (Spearman’s rho = 0.95, *p* < 0.001).

**TABLE 5:** Mean (±SE) pre-oviposition, oviposition, and post-oviposition duration of *Ruspolia differens* reared on different diets.

Diet	Pre-oviposition period (days)	Duration of oviposition (days)	Post-oviposition duration (days)
Diet 1	27.9 ± 2.8^a^	41.7 ± 9.6^a^	7.4 ± 5.0^a^
Diet 2	13.6 ± 0.4^b^	36.4 ± 9.2^b^	0.1 ± 0.1^b^
Diet 3	16.5 ± 1.6^ab^	53.5 ± 7.0^a^	13.6 ± 7.0^a^
Diet 4	13.1 ± 0.8^b^	39 ± 8.1^a^	15.6 ± 3.4^a^
χ^2^	11.11	36.59	14.45
df	3	3	3
p-value	0.011	<0.001	0.002

Note: Mean (±SE) number of days with different superscript letter (s) in the same column are significantly different at *p* < 0.05.

A significant difference was recorded in the post-oviposition duration of *R. differens* fed on various diets (χ^2^ = 14.45, df = 3, *p* = 0.002). The shortest post-oviposition duration occurred in Diet 2 (<1 day), while the longest duration was recorded in *R. differens* fed Diet 4 (16 days) (Table 5).

#### 3.8.2 Effect of diet on fecundity and hatchability

**FIGURE 5:**
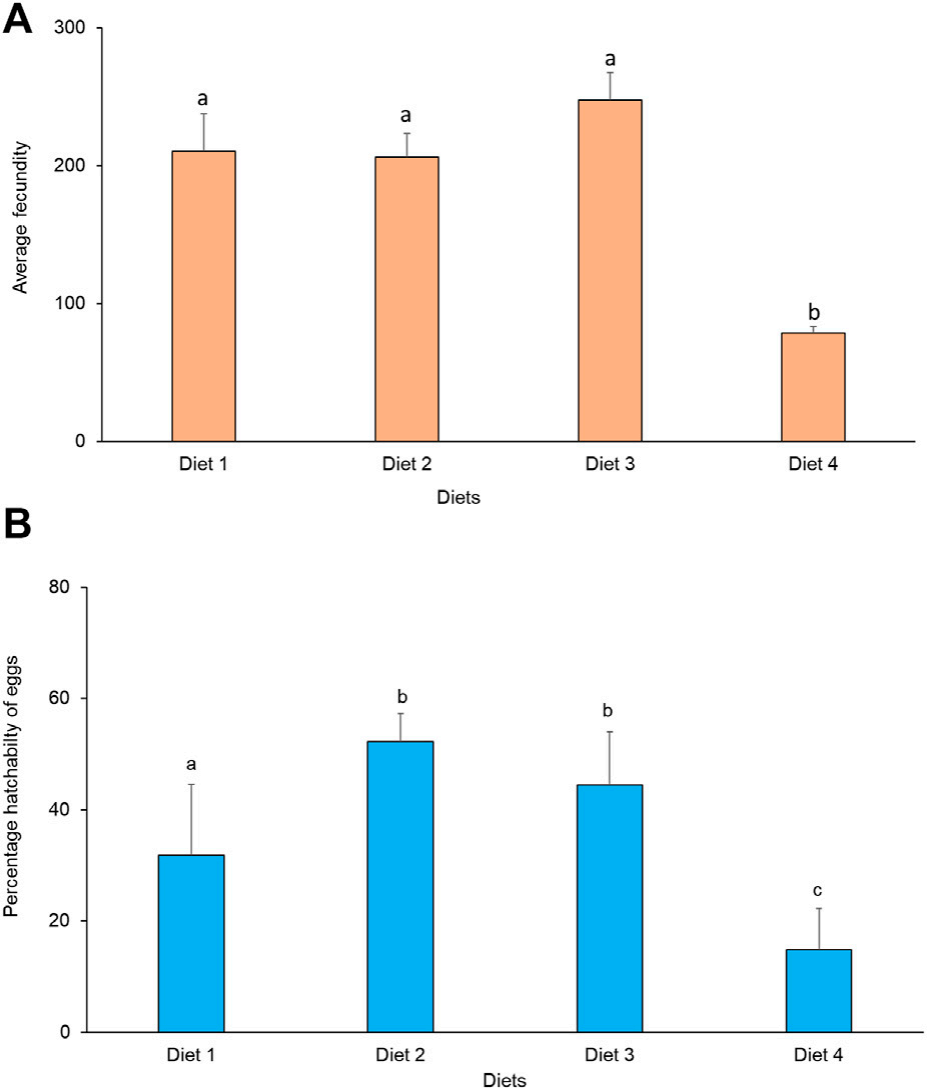
**(A)** Average female fecundity and **(B)** percentage hatchability of eggs of *Ruspolia differens* fed on the different diets. Means capped with different letters differed significantly (Tukey HSD, *p* <0.05).

Average fecundity differed significantly between treatments (χ2 = 11.82, df = 3, *p* = 0.008). The highest fecundity occurred in Diet 3 (248 eggs), while the lowest fecundity was recorded in Diet 4 (77 eggs) (Figure 5A). There was no significant correlation between fecundity and weight in the different diets. A positive significant correlation was observed between fecundity and longevity of *R. differens* fed Diet 2 (Spearman’s rho = 0.78, *p* = 0.007) and Diet 3 (Spearman’s rho = 0.67, *p* = 0.035), while there was no significant correlation when Diet 1 and Diet 4 were used. Percentage hatchability of eggs varied significantly among *R. differens* fed different diets (χ2 = 461.86, df = 3, *p* < 0.001). The highest hatchability percentage of eggs was recorded in Diet 2 (52%), while the lowest hatchability was recorded in Diet 1 (15%) (Figure 5B).

Egg incubation duration did not differ among diets (χ^2^ = 5.92, df = 3, *p* = 0.116). The shortest incubation duration occurred in *R. differens* fed Diet 1 (21 days), while the longest duration was observed in Diet 4 (29 days). Duration of egg eclosion varied significantly among diets (χ^2^ = 129.38, df = 3, *p* < 0.001). The shortest duration of egg eclosion occurred in Diet 4 (20 days), while the longest duration was recorded in Diet 1 (59 days) (Table 6).

**TABLE 6:** Mean (±SE) incubation and egg eclosion duration of *Ruspolia differens* raised on different diets.

Diet	Incubation duration (days)	Duration of egg eclosion (days)
Diet 1	20.7 ± 0.4	59.2 ± 5.7^a^
Diet 2	24.2 ± 0.9	51.9 ± 5.5^a^
Diet 3	23.4 ± 0.7	56.2 ± 8.8^a^
Diet 4	28.8 ± 1.5	19.6 ± 6.1^b^
χ^2^	5.92	129.38
*Df*	3	3
*p*-value	0.116	<0.001

Note: Mean (±SE) number of days with different superscript letter (s) in the same column are significantly different at *p* < 0.05.

### 3.9 Effect of diet on adult longevity

**FIGURE 6:**
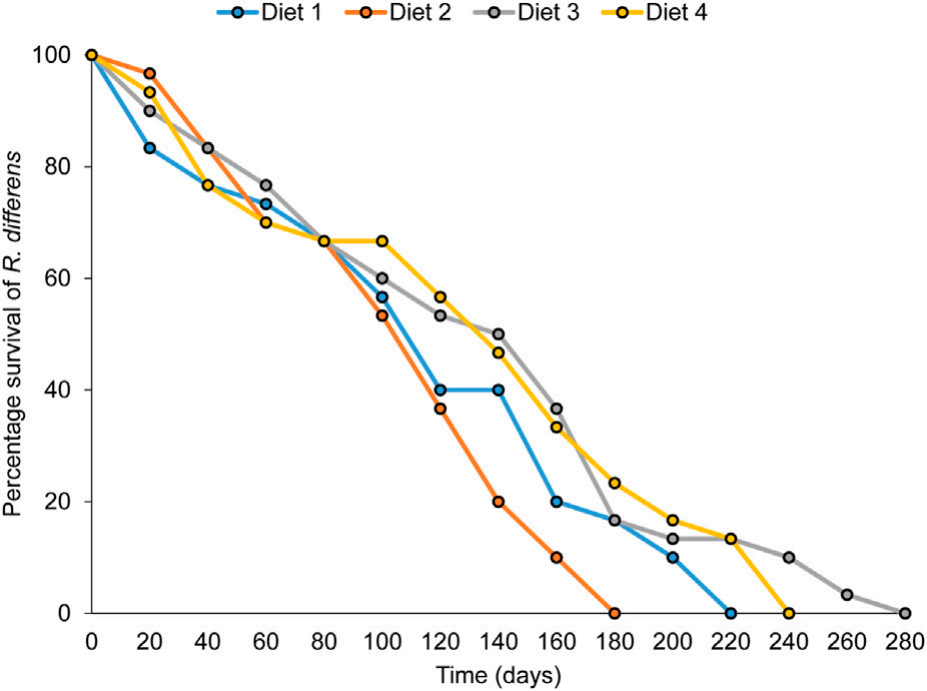
Overall survival rate and longevity of *Ruspolia differens* fed on the various diets.

The overall adult longevity varied significantly among diet types (F_3,76_ = 3.716, *p* = 0.015) (Table 7). The highest longevity occurred in grasshoppers fed Diet 3 (89 days), while the lowest was in *R. differens* fed Diet 2 (51 days). Male and female longevity were not varied (F_1,75_ = 0.134, *p* = 0.715). Male longevity differed significantly among the diets (F _3,36_ = 4.198, *p* = 0.012), with the highest longevity occurring in male *R. differens* fed Diet 3 (97 days). Longevity of female *R. differens* was not different among diets (F _3_, _19_ = 1.8, *p* = 0.181) (Table 7). However, the overall longevity and survival rate of *R. differens* fed Diet 3 was longer compared that fed the various other diets (Figure 6).

**TABLE 7:** Mean (±SE) overall adult longevity and adult longevity by sex of *Ruspolia differens* reared on various diets.

Diet	Mean (±SE) number of days		
	Overall longevity of adults	Male longevity	Female longevity
Diet 1	66.8 ± 10.1^ab^	60.1 ± 11.6^ab^	73.4 ± 16.9^a^
Diet 2	50.5 ± 6.2^a^	51 ± 9.2^a^	50 ± 8.79^a^
Diet 3	88.9 ± 11.2^b^	96.8 ± 8.6^b^	80.9 ± 21^a^
Diet 4	84.7 ± 8.3^b^	89.7 ± 13.4^ab^	79.8 ± 10.3^a^
F-value	3.72	4.20	1.80
Df	3, 76	3, 36	3, 19
*p*-values	0.015	0.012	0.181

Note: Mean (±SE) number of days with different superscript letter (s) in the same column are significantly different at *p* < 0.05.

## 4 Discussion

*Ruspolia differens* has been successfully reared on diverse diets including mixtures of natural host plants, artificial diets, and a combination of artificial diets with host plants (Malinga et al., 2018b; Valtonen et al., 2018; Leonard et al., 2022). Recently, plant by-product diets were shown to provide adequate nutrients for growth and survival of *R. differens* (Sorjonen et al., 2020). Similarly, other insect species such as crickets have been reared successfully on diets comprising by-products of food manufacturing and agriculture (Miech et al., 2016; Sorjonen et al., 2019). However, diets constituted with by-products only may lack key nutritional components essential for the growth and development of insects (Sorjonen et al., 2019). The findings of this study demonstrate that variations in innovative feedstocks containing mixtures of agricultural by-products, MOLP, *ochong’a*, and soybean meal provided sufficient nutrients that influenced the growth, survival, and reproductive performance of *R. differens*. Moringa, *ochong’a*, and soybean are rich in proteins and are increasingly used as an alternative source of proteins for production of livestock feed (Mugo-Bundi et al., 2015; Mukherjee, et al., 2016; Ashour et al., 2020).

*Ruspolia differens* accepted all the diets; however, agricultural by-products (maize and wheat bran) were the most preferred. Wheat bran is among the most preferred feed by *R. differens*, which is an oligophagous species that accepts diverse feed in the absence of its host plants (Malinga et al., 2018a; Valtonen et al*.*, 2018). Diet 3 was the most optimal diet, which significantly reduced developmental time, improved survival above 87%, increased fecundity, and extended longevity of *R. differens*. The addition of MOLP, *ochong’a*, and soybean increased the crude protein, crude fat, and mineral content and digestibility of Diet 3, while the carbohydrate content decreased. A high nutritional value of diets leads to improved growth, development, survival, and reproductive performance of insects (Lindroth et al., 1991).

Development of *R. differens* in the study was faster (57–75 days) than the shortest development period of 100 days and 89 days reported by Malinga et al. (2018) and Leonard et al. (2022), respectively, who reared *R. differens* on diverse diets. A faster development occurred in *R. differens* raised on Diet 3 compared to the other diets, while a slower development occurred in Diet 1. Diet 3 had higher protein, carbohydrate, and mineral (sodium and iron) contents, and higher digestibility compared to Diet 1, which could explain the variations. Rearing of insects on poor diets can result in an increase in larval development time due to unmet nutritional demands (Silva et al., 2009; Kekeunou et al., 2010; Li et al., 2020), which may have been the case in Diet 1. Proteins and carbohydrates play a critical role in the development of insects (Joern and Behmer 1997; Rho and Lee 2014; Roeder and Behmer 2014; Li et al., 2015). Higher crude protein content in diets yielded a shorter development time in crickets *Scapsipedus icipe* (Orthoptera: Gryllidae) and *Acheta domesticus* Linnaeus (Orthoptera: Gryllidae) (Magara et al., 2019; Kuo and Fisher 2022), while high protein and carbohydrate content led to better growth and development rate in some grasshopper species (Malinga et al., 2018). The extremely low content of sodium in Diet 1 (15 times lower than the other diets) and high content of MOLP compared to the other diets could have contributed to the prolonged development. Sodium is critical for the growth and development of vertebrate and invertebrate herbivores with deficiencies, resulting in decreased growth, survival, and reproduction (Peterson et al., 2021). On one hand, excessive use of MOLP is associated with anti-nutritional factors that lower digestibility of diets in livestock (Chadare et al., 2018; El-hack et al., 2018). Although Sorjonen et al. (2020) reported a shorter development time in *R. differens* reared on diets with a protein content between 15% and 22%, the shortest development time occurred in grasshoppers raised on Diet 2 and 3, which had a protein content between 25% and 28%. The male grasshoppers developed faster than the females. This is due to the fact that females have an extra instar where the ovaries develop, and the wings and ovipositor lengthen (Brits and Thrornton, 1981).

*Ruspolia differens* raised on Diet 3 recorded the highest nymphal survival (87%), which was higher than 84% survival recorded when *R. differens* was reared on plant based by-products (Sorjonen et al., 2020) and higher than values recorded when *R. differens* were reared on single and mixtures of their natural host plants, mixed artificial diets, and mixtures of host plants and artificial diet (Malinga et al., 2018b, 2020; Ssepuuya et al., 2018; Leonard et al., 2022). It was, however, lower than 100%, and 96% survival was recorded when *R. differens* were reared individually on food crops (germinated finger millet, fresh maize comb, and sorghum seed head) (Malinga et al., 2022). These differences can be attributed to differences in the nutritional value and diversity of diets. Feeding newly hatched larvae on sufficient food resources results in faster development and a low rate of mortality due to decreased damage during development (Li et al., 2020). Diet 3 seems to have had the optimal quantity of nutrients for the survival of the grasshoppers. Survival of nymphs raised on Diet 2 and 4 that contained higher and lower levels of sodium and iron, respectively, compared to Diet 3, were similar albeit lower, while Diet 1 that contained minimal levels of the minerals had the least nymphal survival. Survival of insects require optimum amounts of sodium and iron, where excess or inadequate amounts of the two minerals results in increased mortality (Hernández et al., 2012; Ferrero et al., 2017; Peterson et al., 2021). According to Sorjonen et al. (2020), no further enhancement in survival was seen when *R. differens* were reared on diets with protein content beyond 17% (Sorjonen et al., 2020). This was contrary to our findings where survival was higher at protein content above 20%. High protein content in diets has been reported to increase mortality in some insects, such as ants (Roeder and Behmer, 2014). Inadequate protein quantity and quality during development increases mortality on immature stages of insects. A significantly lower larval survival was reported on Mediterranean fruit flies, *Ceratitis capitata* Wiedemann (Diptera: Tephritidae) reared on a low-protein diet (Nash and Chapman, 2014) similar to the case in Diet 1. Feeding of *R. differens* on diets with low-protein content and inadequate nutrients results in cannibalism that has been observed in this species and other Tettigonids (Lehtovaara et al., 2017; Sorjonen et al., 2020). It is possible that there was cannibalism among the nymphs of *R. differens* reared on Diet 1; the number of nymphs fed Diet 1 dwindled on a weekly basis although the number of dead nymphs collected from the cages was minimal.

The weight of *R. differens* was higher than their counterparts reared on natural host plants (0.41–0.45 g), comparable to those fed on high protein and carbohydrate diets (0.56 and 0.55 g) and lower than those reared on diets rich in fatty acids (0.64–0.95 g) (Lehtovaara et al., 2017; Rutaro et al., 2018). The wet adult weight of *R. differens* reared on Diet 3 was not different from the wet weight of grasshoppers reared on the other diets. However, higher weights were recorded in nymphs and adult grasshoppers reared on Diet 4, which could have been attributed to the high crude fat and carbohydrate contents of the diet. This was contrary to the findings of other studies where the weight of insects differed with variations in diet quality. The weight of *R. differens* and other grasshopper species increased with an increase in protein content of diets (Sorjonen et al., 2020), but variations of protein content in the study diets did not translate to differences in weight. This could be attributed to the inclusion of MOLP in diets, which has previously been shown to produce a weight similar to conventional commercial feed in poultry (Gadzirayi et al., 2012). Similarly, the addition of sugar to the diet of *Harmonia axyridis* Pallas (Coleoptera: Coccinellidae) led to a significant increase in weight (Li et al., 2020); however, the variations in the quantity of sugar in the diets did not cause significant differences in weight of *R. differens*. Females attained higher weights compared to males fed on different diets similar to findings by Lehtovaara et al. (2017). Generally, male *R. differens* are smaller in size compared to their female counterparts (Matojo and Yarro, 2013). The wet adult weight of the female was comparable across the diets, whereas the male *R. differens* fed Diet 4 recorded significantly higher weights. The high crude fat and carbohydrate content in Diet 4 could have led to an increase in biomass in the male grasshoppers fed on it. The fat content of insects correlates positively with the fat content of their diets; however, the accumulation of lipids in the body of females is utilized in oogenesis and egg maturation (Li et al., 2020).

Slight variations in diets influence reproductive performance of insects (Du et al., 2015). The mean pre-oviposition duration recorded in Diet 3 did not differ from the other diets. The pre-ovipostion period recorded in Diets 2, 3, and 4 were within a similar range with 16 days previously reported (Brits and Thrornton, 1981); however, *R. differens* fed Diet 1 took almost twice as much time before laying eggs. This was probably due to the low nutrients contained in Diet 1. A prolonged pre-oviposition time is associated with poor nutrient quality of diets that delays oocyte development. Development of oocytes is under endocrine control that is triggered by correct nutrient levels in food (Ahmad and Nabi, 2012). Oocyte development in *Oxya japonica* Thunberg (Orthoptera: Acrididae) was affected by the nutritional requirements of the insect and the chemical composition of food (Ahmad and Nabi, 2012), while addition of sugar to diet significantly shortened the pre-oviposition time in *H. axyridis* (Li et al., 2020). The pre-oviposition time of mites and ladybirds decreased with different diet mixtures, which further corroborate these findings (Bonte et al., 2010; Muñoz-Cárdenas et al., 2014). Additionally, laying late is part of a survivorship strategy to increase longevity in female and chances of encountering more quality diet before oviposition (Hatt and Osawa, 2021). This was demonstrated in Diet 1, which recorded high female longevity despite delayed oviposition. The mean oviposition duration recorded in the study was much higher than the 32 days that was previously reported for *R. differens* (Brits and Thrornton, 1981). The grasshoppers fed Diet 3 laid eggs over a significantly longer duration compared to other diets. Longer duration of oviposition is associated with high protein content in diets (Magara et al., 2019).

The highest fecundity of *R. differens* was comparable to the highest fecundity reported when the grasshopper was raised on an artificial diet mixture with six substrates (Malinga et al., 2018b); but it was 5.5 times higher than fecundity of *R. differens* reared on artificial diet mixed with their host plants (Leonard et al., 2022). Variations in fecundity in phytophagous insects are associated with variations in qualitative and quantitative amounts of nutrients in host plants (Roy and Barik, 2012b). It is possible that the differences arose from variations in the quality of diets used in the various studies. The fecundity of *R. differens* fed Diet 3 was higher but comparable to diets 1 and 2. The difference could be due to protein:sugar ratio of the diets. Diet 1, 2, and 3 were more protein-biased compared to Diet 4. Egg production in phytophagous insects was better on balanced or slightly protein-biased diets (Roeder and Behmer, 2014). A high protein-to-carbohydrate ratio in diet results in high egg production due to the substantial protein investment required for oogenesis in insects (Kim et al., 2020). The low egg production in grasshoppers fed Diet 4 could also be due to high carbohydrate content that was almost two times higher compared to the other diets. The egg production rate in the grasshopper *Melanoplus sanguinipes* Fabricius (Orthoptera: Acrididae) displayed a negative response to increased carbohydrate (Joern and Behmer, 1998). This was, however, contrary to findings on *H. axyridis* where egg production increased with an increase in sugar content in the diet (Li et al., 2020). Although the diversification of diet has been associated with high fecundity, grasshoppers fed Diet 4, which was most the diversified in this study recorded the least number of eggs (Malinga et al., 2018). It is probable that the presence of MOLP in diets 1, 2, and 3 contributed to their better performance than Diet 4. Moringa leaves are rich in vitamins, minerals, and essential amino acids, which may have led to a higher fecundity in the diets with MOLP (Sodamade et al., 2017; Abbas et al., 2018). The inclusion of different nutrient classes in the diets of the Queensland fruitfly, *Bactrocera tryoni* Froggatt (Diptera: Tephritidae), resulted in substantial egg production (Fanson and Taylor, 2012a). There is, however, need for more work to determine the effects of supplementation of diets MOLP on the reproductive performance of edible grasshoppers. There was no correlation between weight and fecundity in the grasshoppers fed on the different diets. This was contrary to the findings of other studies that recorded a positive correlation between weight and fecundity. This was probably due to the limited variations in wet weights of adult female *R. differens* raised on the study diets. In the grasshopper *Ageneotettix deorum* Scudder (Orthoptera: Acrididae) and the cricket *S. icipe*, fecundity correlated positively with female weight when they were reared on different diets (Joern and Behmer 1997; Magara et al., 2019). Large-sized female insects have better reproductive performance compared to smaller ones (Hagen, 1962); however, the weight of adult female *R. differens* raised on the study diets was comparable.

Egg incubation duration was longer than in previous studies that assessed suitability of diverse egg hatching conditions (Ssepuuya et al., 2018; Egonyu et al., 2021; Leonard et al., 2021). The performance of the eggs was, however, not linked to effects of diets, which could explain the differences. The incubation period was shorter for *R. differens* raised on diets 1 and 3 that for controls. Variations in nutrient composition of diets lead to differences in incubation period of insects (Li et al., 2020), where diets with limiting nutrients delay eclosion (Adler et al., 2013). This was contrary to our findings where egg incubation across diets was not consistent with their nutrient composition.

Hatchability percentage was higher in the diets fortified with MOLP compared to control. The hatchability percentage of eggs recorded in Diet 3 was comparable to that in Diet 2 but higher than those in diets 1 and 4. Higher egg hatchability is correlated with high protein content in cricket *S. icipe* (Magara et al., 2019); however, Diet 4, which had a higher protein content, yielded a lower percentage of hatchability compared to Diet 1 that had the least protein content. Reduced hatchability in *Spodoptera exigua* was associated with irrational nutrition (Li et al., 2020). It is possible that addition of MOLP to diets accorded a reproductive advantage to the grasshopper based on the other nutrients obtained from the plant, which may have resulted in the large disparity in the number of eggs hatched between the MOLP-fortified diets and the control. Previously, supplementation of poultry diet with Moringa increased hatchability of eggs (Ashour et al., 2020), which may have been the case in this study. Many insects reared on artificial diets lose the ability to adapt and reproduce resulting into lower fertility (Li et al., 2020). This could explain the low fecundity and percentage hatchability that was observed in Diet 4. It is possible that this diet has a limiting effect on the reproductive performance of the grasshopper. However, there is need for further investigation before such a generalization can be made.

Longevity of adult *R. differens* was similar to longevity recorded by Malinga et al. (2018) and twice as long as the value recorded by Leonard et al. (2022). The highest adult longevity was recorded in Diet 3, which was comparable to Diet 4, whereas Diet 2 had the lowest longevity. The balance between protein and carbohydrate in diets is a key determinant of the relationship between diet and longevity. Excessive consumption of proteins relative to carbohydrates results in a shorter life span in insects with high mortality rates reported in cockroaches, crickets, flies, ants, and bees when confined to diets with higher protein contents relative to their requirements (Dussutour and Simpson, 2012). On the other hand, the reduction of protein to the carbohydrate ratio is associated with increased life span in many taxa (Fanson and Taylor, 2012b). Despite this evidence, it is difficult to attribute the differences observed in the study to variations in carbohydrate and protein contents of diets. The protein and carbohydrate content of Diet 3 and Diet 2 were comparable, but the average longevity recorded in Diet 3 was 1.7 times higher than that in Diet 2. The longevity recorded in Diet 1 that had a lower protein and carbohydrate content than all the diets was equally 1.3 times higher than Diet 2. These differences may have been influenced by micronutrients that were present in the diets. Micronutrients are beneficial for insects and influence their survival in diverse ways (Kuo and Fisher, 2022); however, there is a need for further investigation before a generalization is made. The longevity of male and female grasshoppers raised on the different diets was comparable. This was similar to findings of Magara et al. (2019) but contrary to studies on *Diacrisia casignetum* Kollar (Lepidoptera: Arctiidae), where female longevity was longer than that for males (Roy and Barik, 2012a). The findings were inconsistent with the outcome of the study conducted on *B. tryoni*, which demonstrated that alteration of diet decreased female longevity (Fanson and Taylor, 2012a). Female longevity was positively correlated to fecundity in Diet 2 and Diet 3. This was contrary to reports that organisms cannot maximize fecundity and life span on a single diet due to the nutritional requirements of the two traits that cannot be capitalized concurrently (Kim et al., 2020). However, it was in tandem with insights from nutritional geometry studies that have suggested that the ratio of macronutrients that maximize reproduction is not the same one that minimize life span (Adler et al., 2013). This tradeoff could have occurred in Diet 4 where fecundity was very low despite the high female longevity.

## 5 Conclusion

This study provides the first report on use of low-cost by-products of maize and wheat processing industries fortified with MOLP to formulate an optimal cheap diet for improved mass production of *R. differens*. Diet 3 is the most optimal diet, which significantly reduced the developmental time of *R. differens*, improved survival above 87%, increased fecundity and hatchability, and extended the longevity of *R. differens* to enable easy propagation of future mass production strategies compared to previous studies. The efficiency of Diet 3 was attributed to the high nutrient content, which is associated with improved growth and reproductive performance in insects. The cost–benefit ratio and return of investment in the diet make it a suitable and promising alternative, which would be affordable to everyone including the resource-poor and vulnerable segment of the communities where *R. differens* is widely consumed. Further optimization of this diet would enable year-round mass production of *R. differens* to overcome challenges and bridge the gap of unsustainable dependence on erratic seasonal swarms of these grasshoppers. Further studies are recommended to establish the impact of Diet 3 on the nutritional quality and sensory properties of *R. differens* for improved nutrition and health of consumers.

## Data Availability

The original contributions presented in the study are included in the article/Supplementary Material; further inquiries can be directed to the corresponding authors.
